# Reframing sorghum biofortification: multi-trait nutritional dissection of Sudanese wild germplasm reveals extractability-driven breeding targets

**DOI:** 10.3389/fpls.2026.1888511

**Published:** 2026-07-30

**Authors:** Layla Kamal, Khitma A. Sir Elkhatim, Tagwa M. A. Mohammed Alkhair, Manhal Gobara Hamid, Mohammed Elsafy, Tilal Abdelhalim

**Affiliations:** 1 Biotechnology and Biosafety Research Center, Agricultural Research Corporation, Khartoum North, Sudan; 2 College of Agricultural Studies, Sudan University of Science and Technology, Khartoum North, Sudan; 3 Department of Plant Breeding, Swedish University of Agricultural Sciences (SLU), Alnarp, Sweden; 4 Geo-Biosphere Interactions, Department of Geosciences, University of Tuebingen, Tuebingen, Germany

**Keywords:** antinutritional factors, grain quality traits, heritability, micronutrient extractability, multivariate analysis, wild germplasm

## Abstract

**Introduction:**

Sorghum is a cornerstone of food security in Sudan, yet sorghum-based diets contribute to widespread micronutrient deficiencies. Conventional biofortification has focused on raising grain mineral concentrations, overlooking the antinutrients and matrix factors that govern actual nutrient absorption. We hypothesized that Sudanese wild sorghum, encompassing a deeper allelic pool than cultivated lines, harbors decoupled variation in mineral density, extractability, and protein quality that could redirect breeding toward functional nutritional outcomes.

**Methods:**

A panel of 70 wild accessions, representing six taxa collected across diverse Sudanese agro-ecologies, was advanced through three single-seed-descent cycles and evaluated in a replicated alpha-lattice trial for total and HCl-extractable Fe and Zn, phytate, and soluble protein.

**Results and discussion:**

Substantial variation was detected across traits, with total Fe ranging from 2.65 to 8.20 mg/100 g and Fe extractability reaching 44.12%, often in genotypes with modest total Fe levels. Total Zn (0.25–0.81 mg/100 g) and Zn extractability (up to 57.37%) likewise showed independent distributions, while phytate (210.37–380.21 mg/100 g) varied largely independently of HCl-extractable Fe. Principal component analysis explained 55.4% of the variance and separated mineral accumulation, extractability, and protein functionality into orthogonal trait domains. High broad-sense heritability (0.83–0.99) and substantial genetic advance (GAM up to 54.19%) indicated strong selection potential. Elite accessions, notably HSD14541 and HSD14468, combined low phytate with high mineral extractability and protein solubility. These findings establish wild Sudanese sorghum as a strategic genetic resource and reposition in vitro mineral accessibility (estimated as HCl-extractability), rather than concentration alone, as a breeding target.

## 1 Introduction

The eastern Sahel and the Sudanese Nile corridor are recognized as primary centers of sorghum (*Sorghum bicolor*) domestication. Archaeobotanical evidence dates the emergence of domesticated forms from wild progenitors to the 3rd–2nd millennium BCE (Capasso et al., 2024; Fuller and Stevens, 2018; Venkateswaran et al., 2019; Winchell et al., 2018). Key sites, including Jebel Moya, KG23 at Khashm el Girba, and Mahal Teglinos K1, document the co-occurrence of wild and domestication-related traits, most notably the increasing frequency of non-shattering spikelets, through ceramic impressions, phytoliths, flotation remains, and micro-computed tomography (Barron et al., 2020; Gregory et al., 2023; Winchell et al., 2017). Together, these records indicate a protracted domestication of wild sorghum in eastern Sudan.

This protracted domestication, followed by westward dispersal across Africa, generated the broad genetic diversity now distributed across the crop and its wild relatives (Beldados et al., 2018; Beldados and Ruiz-Giralt, 2023; Cruet-Burgos et al., 2023; Weerasooriya et al., 2018). Wild relatives are of particular value because they retain allelic diversity that was partially lost during domestication, and ongoing gene flow among wild, weedy, and cultivated pools maintains a broad reservoir of adaptive variation (Mutegi et al., 2010, 2012; Ohadi et al., 2017; Sagnard et al., 2011; Smith et al., 2019). Genomic and pangenomic analyses indicate that this reservoir includes alleles relevant to stress tolerance and grain nutritional quality, identifying wild sorghum as a promising source for trait improvement (Morris et al., 2026; Ruperao et al., 2021).

Beyond its evolutionary significance, sorghum is a staple of food and feed systems across the semi-arid tropics, valued for its drought tolerance and adaptation to the marginal, rainfed environments where other cereals fail (Boukrouh et al., 2021, 2023). As a food grain it supports diverse applications, including use as a rice substitute, gluten-free flour, syrup, and plant-based beverages, and contributes appreciable protein, fibre, and bioactive compounds, while its gluten-free nature makes it suitable for individuals with celiac disease (Tan et al., 2024). Sorghum grain and fodder are equally important in livestock systems, where they serve as competitive alternative feed resources that sustain meat and milk performance: sorghum grain can replace conventional concentrates in fattening diets without compromising growth or carcass quality (Boukrouh et al., 2024), and improved low-lignin fodder cultivars can enhance milk yield in ruminants (Dey et al., 2026). This dual role in human and animal nutrition, particularly in food-insecure regions, makes improving the nutritional quality of sorghum grain a breeding priority.

Conventional biofortification has predominantly targeted higher iron (Fe) and zinc (Zn) concentrations in staple crops (Ibrahim et al., 2021). However, the nutritional benefit of higher grain mineral content is ultimately limited by the fraction the body can absorb, because phytic acid (phytate) chelates Fe and Zn and reduces their absorption (Carvalho and Vasconcelos, 2013; Ciccolini et al., 2017; Garcia-Oliveira et al., 2018; Raboy, 2020). It is therefore important to distinguish three related concepts: mineral concentration (the total amount in the grain), bioaccessibility (the soluble fraction released from the matrix and potentially available for uptake), and bioavailability (the fraction actually absorbed and utilized, which requires Caco-2, animal, or human assays to measure). Because such absorption assays are not tractable for screening large germplasm panels, the present study uses dilute-HCl extractability as an *in vitro* estimate of the soluble, potentially bioaccessible mineral fraction; it indexes solubility under simulated gastric acidity and does not, by itself, measure intestinal absorption (Jha and Warkentin, 2020; Shahzad et al., 2014). Used in this way, HCl extractability provides a high-throughput, breeding-relevant proxy that complements total mineral measurement.

These factors are interconnected within the grain matrix: mineral density sets the upper limit of nutritional supply, phytate governs how much of that supply remains soluble and absorbable, and protein contributes both nutritional value and matrix interactions that further modulate mineral solubility (Cominelli et al., 2020; Gupta et al., 2015; Sharma et al., 2022; Zhang et al., 2022). In this study, protein functionality refers specifically to protein solubility, the proportion of grain protein extractable into buffer, which serves as a practical indicator of protein quality and of the protein–matrix behavior relevant to mineral release, and is more readily measured across large panels than digestibility or amino acid composition. Evaluating mineral concentration, mineral extractability, phytate, and protein solubility together therefore provides a more complete picture of nutritional value than mineral concentration alone.

The wild gene pool of sorghum remains underexplored for micronutrient density and its interactions with antinutrients, particularly in African centers of diversity. Abdelhalim et al. (2019) characterized Sudanese wild sorghum for grain Fe, Zn, phytate, and tannin contents, establishing that substantial variation exists. However, these traits have rarely been assessed simultaneously with *in vitro* mineral accessibility and protein solubility, and selection on mineral concentration alone may inadvertently favor genotypes with poor functional nutrition owing to high phytate (Ram et al., 2020). The present study differs from this earlier work by moving from single-trait characterization to an integrated, multi-trait evaluation that jointly quantifies total minerals, HCl-extractable minerals, phytate, and protein solubility in the same panel, allowing genotypes to be prioritized on functional nutritional profiles rather than concentration alone. Advances in quantitative trait locus mapping and marker-assisted selection further provide opportunities to improve Fe, Zn, and protein traits jointly within a unified breeding framework (Garcia-Oliveira et al., 2018; Ghafar et al., 2025).

This study, therefore, aimed to provide a comprehensive, integrated phenotypic evaluation of Sudanese wild sorghum that jointly quantifies total minerals, *in vitro* mineral accessibility, phytate, and protein solubility, in order to identify low-phytate, accessibility-enhanced genotypes suitable for nutrition-focused pre-breeding. A panel of 70 wild accessions was phenotyped, and genotypes combining low phytate with elevated HCl-extractable minerals and high protein solubility were prioritized as candidates for further validation.

## 2 Materials and methods

### 2.1 Plant materials and experimental setup

Wild sorghum genotypes (n = 70) representing six taxa, *Sorghum bicolor* subsp. *verticilliflorum*, *S. aethiopicum*, *S. purpureosericeum*, *S. halepense*, *S. virgatum*, and *Sorghum* spp., were collected from 47 geo-referenced locations across Sudan during 2016–2017 and deposited at Agricultural Plant Genetic Resources Conservation and Research Center (APGRC) of the Agricultural Research Corporation (ARC). Taxonomic identification of accessions followed APGRC passport data, based on panicle and spikelet morphology, shattering habit, and glume characteristics per the IBPGR sorghum descriptors. Voucher specimens are maintained at the APGRC under accession codes (HSD series). Accessions that could not be resolved to a described taxon are designated *Sorghum* sp. and were retained as an unassigned group rather than excluded.

The number of accessions per taxon was unbalanced (*S. bicolor* subsp. *verticilliflorum* dominated the panel, while *S. virgatum* and *Sorghum* sp. were represented by ≤ 5 accessions each); this imbalance reflects the natural distribution of taxa in Sudan and is acknowledged as a limitation for taxon-level interpretations.

**Figure 1:**
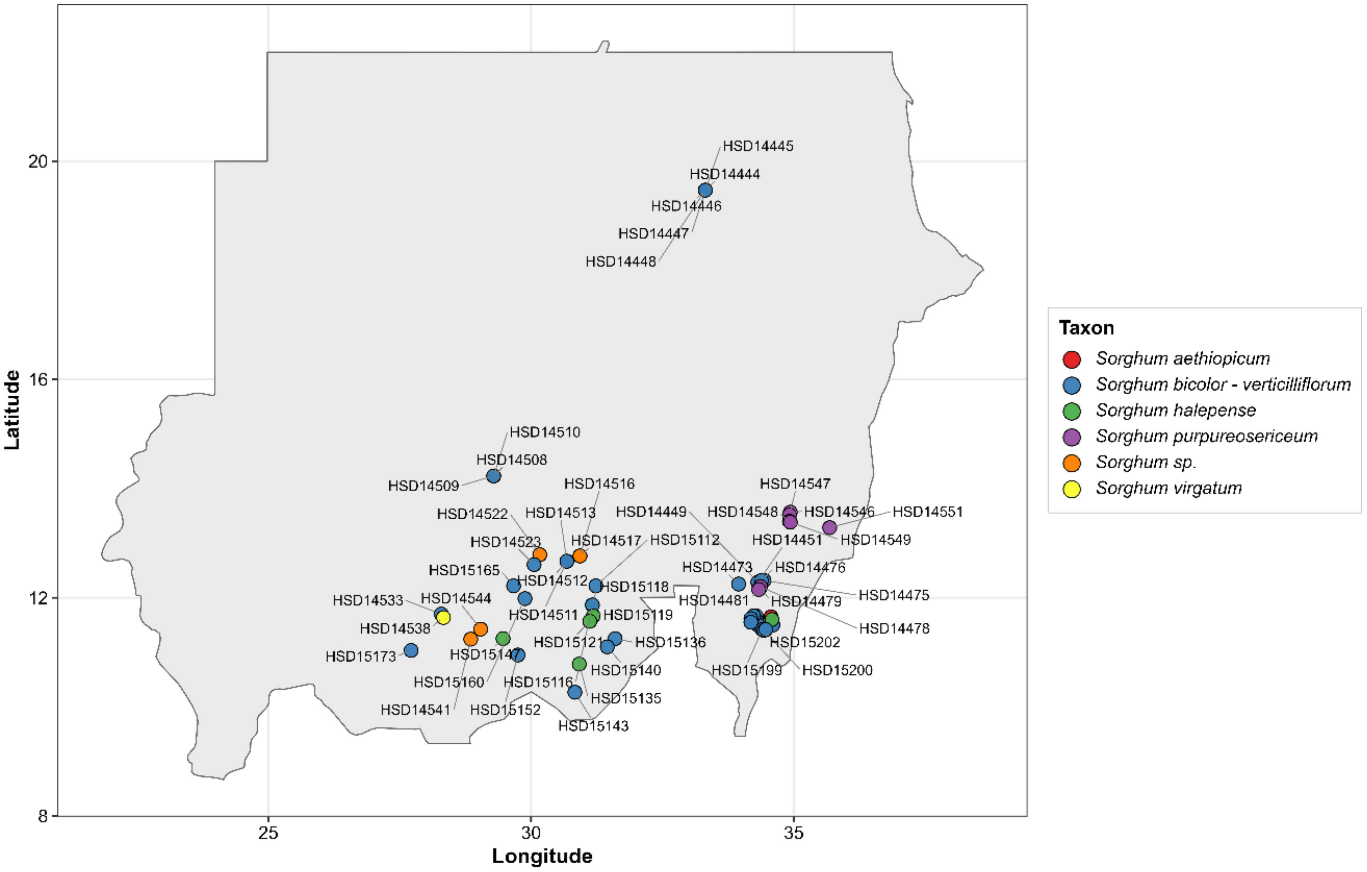
Distribution of wild sorghum germplasm across environmental gradients in Sudan. Wild sorghum accessions (n = 70; 47 sites, 2016–2017) span six states across semi-arid to sub-humid climates.

Sampling covered six states (Northern, Blue Nile, Gedaref, North Kordofan, South Kordofan, and West Kordofan) (**Figure 1**), spanning ~10.3–19.5° N and 27.7–35.7° E, with elevations ranging from 440–690 m above sea level. These domains include the sub-humid Blue Nile highlands and riverine systems, the transitional lowland–foothill landscapes of Gedaref, the semi-arid plains and plateaus of the Kordofan region, and the arid region of Northern Sudan. Points on the map represent individual collection sites, with symbol groups indicating repeated sampling at these locations (**Figure 1**).

Owing to their lax panicle architecture and high natural outcrossing rate, the wild accessions were advanced using the single-seed descent (SSD) method over three consecutive generations (2018–2021) to increase homozygosity and improve within-accession uniformity prior to phenotypic evaluation. In each generation, panicles were enclosed in paper bags before anthesis to enforce self-pollination, prevent cross-fertilization, and protect developing seeds from bird damage. One selfed seed from each plant was randomly selected and advanced to the subsequent generation following the SSD procedure. Because three generations of SSD are insufficient to achieve complete homozygosity in highly outcrossing wild germplasm, residual heterozygosity is expected to remain. Consequently, the resulting accessions should be regarded as partially inbred SSD-derived lines rather than fully fixed genotypes.

The 70 Sudanese wild sorghum genotypes were sown in an alpha-lattice design with three replicates (210 plots). Each replicate was subdivided into five incomplete blocks of 14 plots, with genotypes randomly allocated to the blocks at the Biotechnology and Biosafety Research Center, Agricultural Research Corporation (ARC), Sudan (Khartoum North, Shambat) during the 2022–2023 season. Each accession was grown in 4 m-long rows with 30 cm between hills and 80 cm between rows, resulting in approximately 13 hills per plot. Two plants were maintained per hill throughout the growing season. To minimize border effects, three competitive plants were sampled from the central portion of each plot for subsequent grain analyses. Grain harvested from the three sampled plants was bulked on a plot basis prior to milling and laboratory analyses. Due to their wild nature, the panicles were threshed, and for some accessions, the glumes of the harvested grains were carefully removed with forceps. For the mineral and nutritional analyses, the grains were carefully cleaned, free of foreign materials, and milled with a laboratory grinder fitted with a 0.4 mm screen to achieve a uniform particle size. The resulting flour was stored in airtight plastic zip bags at 4 °C until chemical analysis.

### 2.2 Determination of total minerals

The total iron (Fe) and zinc (Zn) contents were determined according to the method described by Mawouma et al. (2023), with minor modifications. Grain samples were finely ground using a grinder fitted with stainless-steel components. To minimize trace-metal contamination, all glassware and digestion vessels were soaked in 10% (v/v) nitric acid (HNO^3^), thoroughly rinsed several times with deionized water, and air-dried before use. Briefly, 2.0 g of the finely ground sorghum grain sample was weighed into porcelain crucibles and incinerated in a muffle furnace at 500 °C for 3 h until a constant greyish-white ash was obtained. After cooling in a desiccator, the ash was dissolved in 5 mL of 1 N nitric acid (HNO^3^) and filtered through Whatman No. 1 filter paper into a 100 mL volumetric flask. The volume was adjusted to 100 mL with deionized water (Sahrawat et al., 2002).

The resulting digest was analysed for Fe and Zn using flame atomic absorption spectrophotometry (AAS) (PerkinElmer Model 2380, Norwalk, CT, USA) equipped with an air–acetylene flame. Iron and zinc were quantified at wavelengths of 248.3 and 213.9 nm, respectively, using the manufacturer’s recommended instrumental settings (Ni et al., 2007). Calibration curves were prepared using Fe and Zn standard solutions. Analytical quality was monitored by including reagent blanks with each analytical batch, and calibration curves showed excellent linearity (R² > 0.99). All samples were analysed in triplicate, and analytical precision was maintained at a coefficient of variation (CV) below 5%. Results were expressed on a dry-weight basis (mg/100 g).

### 2.3 HCl extractability of minerals (*in vitro* mineral accessibility)

*In vitro* mineral accessibility was estimated as the dilute-HCl-extractable fraction of Fe and Zn, following Khetarpaul and Chauhan (1989a, 1989b) and Mahajan and Chauhan (1988) with minor refinements. Finely ground grain (1 g) was extracted with 10 mL of 0.03 N HCl and incubated at 37 °C for 1 h under continuous agitation to simulate mild gastric solubilization. The suspension was filtered (Whatman No. 1) at 37 °C, and the filtrate, representing the acid-soluble mineral fraction, was oven-dried at 60 °C and dry-ashed/acid-digested using the same procedure as for total minerals, so that extractable and total Fe and Zn were quantified by identical AAS calibration and were directly comparable.

HCl extractability (%) was defined as follows:


$$HCl\;extractability(\%)=\frac{mineral\;extractable\;in\;0.03\;N\;HCl}{total\;mineral}\times 100$$

### 2.4 Phytate content

Phytate (phytic acid) content was determined using the Wade reagent colorimetric method described by Gao et al. (2007), with slight modifications. Briefly, approximately 50 mg of finely ground sample was extracted with 10 mL of 0.66 N HCl at ambient temperature for 60 min, with intermittent shaking to ensure complete solubilization. The extract was centrifuged at approximately 1, 000 × g (3000 rpm) for 10 min at room temperature to obtain a clear solution. An aliquot of 3 mL of the supernatant was mixed with 1 mL of Wade reagent (0.03% FeCl^3^·6H_2_O and 0.3% sulfosalicylic acid prepared in distilled water). The mixture was vortexed and centrifuged again at approximately 1, 000 × g (3000 rpm) for 10 min to remove turbidity.

The absorbance of the resulting solution was measured at 500 nm using a UV–visible (UV–VIS) spectrophotometer (UV–VIS PD-303). This wavelength corresponds to the maximum absorbance of the Fe³^+^–phytate complex formed under the assay conditions, enabling quantitative determination. A calibration curve was constructed using sodium phytate standards, and the phytate content was calculated and expressed as mg phytic acid per 100 g dry weight.

### 2.5 Determination of protein solubility

Protein was isolated using a method adapted from Afify et al. (2012), with minor modifications to enhance the extraction efficiency and analytical reproducibility in sorghum matrices. Initially, 1 g of wild sorghum flour was homogenized with 25 mL of 1 M sodium phosphate buffer (pH 7.0; NaH_2_PO_4_/Na_2_HPO_4_) to maintain protein stability during extraction. The mixture was agitated for 12 h at 150 rpm (ambient) to maximize protein release, following the extended-extraction protocol of Afify et al. (2012). The slurry was centrifuged at approximately 1, 000 × g (3000 rpm) for 10 min, and the supernatant (buffer-soluble protein fraction) was assayed by the method of Lowry et al. (1951). Soluble protein was expressed as g/100 g DW.

Briefly, 1 mL of the supernatant and 5.5 mL of alkaline copper solution (0.5% CuSO_4_·5H_2_O in 1% potassium sodium tartrate with 2% Na_2_CO^3^ in 0.1 N sodium hydroxide, at a 50:1 (v/v) ratio) were mixed thoroughly and incubated at room temperature for 10 min to allow the formation of the protein–copper complex. Subsequently, 0.5 mL of Folin–Ciocalteu reagent (diluted 1:1 (v/v) with distilled water) was added, and the mixture stood in the dark for 30 min to develop the chromophore, read at 660 nm using a UV/visible spectrophotometer (UV–VIS PD-303 UV), with a blank as a reference. Bovine serum albumin was used as the standard protein to construct a calibration curve and quantify the protein. Soluble protein was expressed as g/100 g DW sample to ensure comparability with established cereal protein solubility studies.

### 2.6 Statistical analysis

Analyses were performed in R (R Core Team, 2024). For each trait, genotype effects and adjusted means were estimated from a linear mixed model fitted by REML that accounted for the alpha-lattice structure: trait ~ genotype + (1 | replicate) + (1 | replicate: block), with genotype as a fixed effect for mean estimation and as a random effect for variance-component estimation. Incomplete blocks were thus modeled as random within replicates, recovering inter-block information and improving the precision of genotype comparisons relative to a model ignoring the design.

Genotypic (σ²g) and residual (σ²e) variances were obtained from the random-genotype model. Phenotypic variance was computed on an entry-mean basis as σ²p = σ²g + σ²e/r, where r is the number of replicates. Broad-sense heritability was estimated as H² = σ²g/σ²p. Genotypic and phenotypic coefficients of variation (GCV, PCV), genetic advance (GA), and genetic advance as a percentage of the mean (GAM) were derived following standard quantitative-genetic formulations (Burton and DeVane, 1953; Gomez and Gomez, 1984), with GAM emphasized over GA for cross-trait comparison because GA is scale-dependent.

Pairwise trait associations were quantified by Pearson correlation coefficients and visualized as hierarchically clustered, color-coded matrices with *corrplot* (Wei and Simko, 2024). Multivariate structure was further explored using principal component analysis (PCA) with *FactoMineR* (Lê et al., 2008), and biplots, scree plots, and variable-contribution charts were produced with *factoextra* (Kassambara and Mundt, 2016). For multi-trait visualization, raw values were standardized to z-scores (mean = 0, SD = 1) using the base *scale ()* function, and hierarchical clustering of genotypes was performed on Euclidean distances with complete-linkage agglomeration and rendered using *ComplexHeatmap* (Gu and Hübschmann, 2022).

To integrate multi-trait nutritional performance, a directional standardization was applied: z-scores for phytate were sign-reversed (so that lower phytate yielded a higher score), while all other traits retained their natural direction. A composite nutritional index was then computed as the unweighted mean of the 6-directional z-scores per genotype, enabling ranking and identification of superior entries. These rankings were displayed using faceted bar charts and z-score heatmaps generated with *ggplot2* (Wickham, 2016) and assembled with *patchwork* (Pedersen, 2024). Radar plots comparing the top-performing genotypes were produced with *fmsb* (Nakazawa, 2018) following min–max normalization of trait values to a 0–1 scale to ensure intuitive comparative interpretation.

## 3 Results

**Figure 2:**
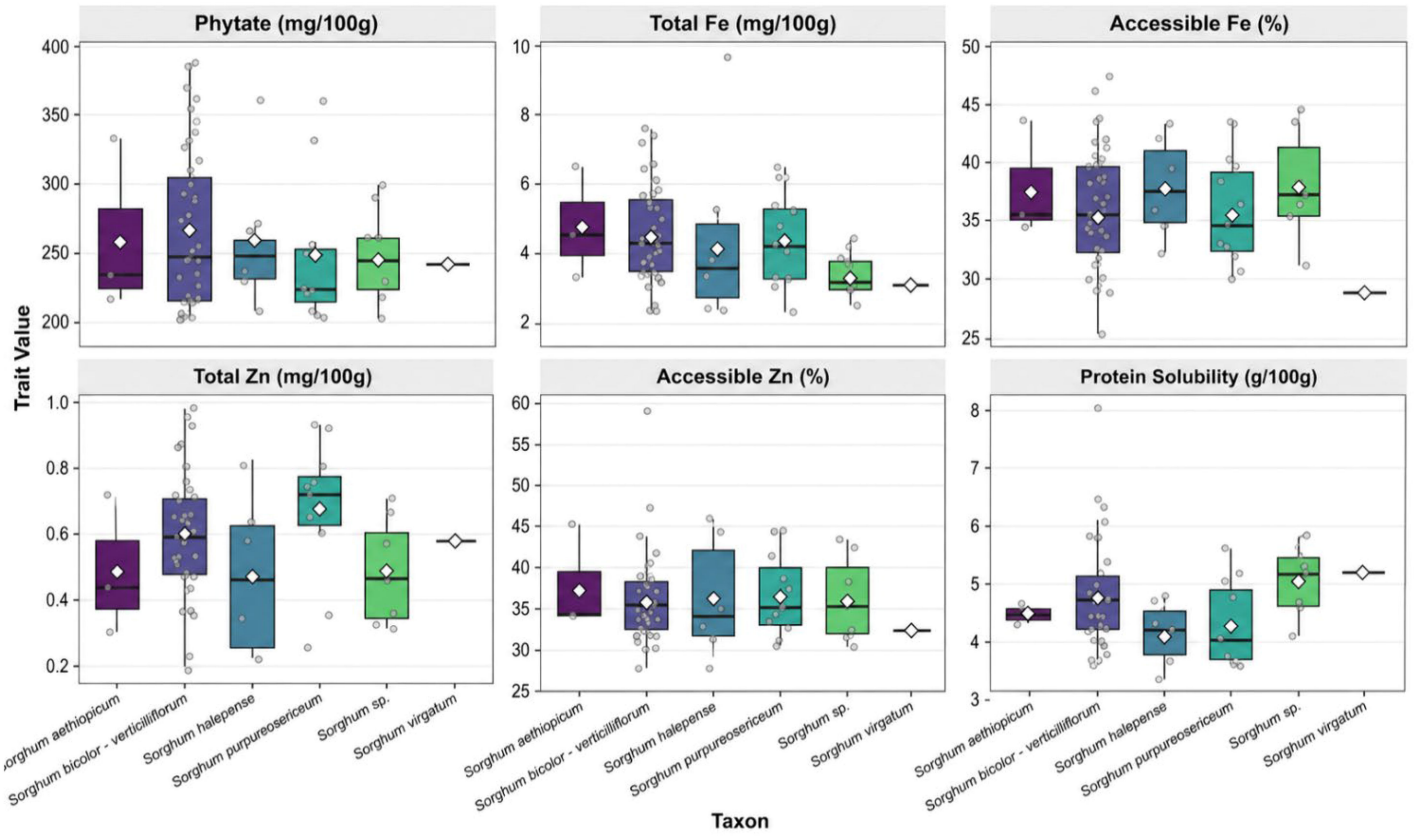
Interspecific variation in nutritional traits among the Sudanese wild sorghum taxa. Boxplots showing the distributions of extractable Fe (%), extractable Zn (%), phytate (mg/100 g), protein solubility (g/100 g), total Zn (mg/100 g), and total Fe (mg/100 g) across sorghum taxa. Boxes represent interquartile ranges, horizontal lines indicate the median, and points denote individual observations.

Taxon-level means for the six nutritional traits are summarized in **Table 1** and **Figure 2**, with the number of accessions per taxon indicated. The panel was strongly unbalanced: *Sorghum bicolor* subsp. *verticilliflorum* was represented by 40 accessions, whereas *S. virgatum* was represented by a single accession, which limits the resolution of taxon-level comparisons. When tested across taxa represented by more than one accession, the taxon effect was not statistically significant for any trait (one-way ANOVA, all p ≥ 0.17; Kruskal–Wallis, all p ≥ 0.17). Some descriptive tendencies were apparent, for example, slightly higher Fe extractability in *Sorghum* sp. and *S. aethiopicum*, and higher protein solubility in S. virgatum and *Sorghum* sp., but these differences fell within the range of among-genotype variation and were not supported statistically. Taxon-level trends should therefore be regarded as preliminary, and the substantive nutritional variation in this panel was expressed at the genotype rather than the taxon level, as detailed below.

**Table 1:** Taxon-level means (± SE) for six nutritional traits in the wild Sudanese sorghum panel, with the number of accessions per taxon. The taxon effect was not statistically significant for any trait (one-way ANOVA across taxa with n ≥ 2).

Taxon	n	Phytate (mg/100 g)	Total Fe (mg/100 g)	HCl-extractable Fe (%)	Total Zn (mg/100 g)	HCl-extractable Zn (%)	Soluble protein (g/100 g)
*S. bicolor subsp. verticilliflorum*	40	269.4 ± 8.7	4.73 ± 0.20	35.3 ± 0.7	0.54 ± 0.02	36.1 ± 0.8	4.25 ± 0.11
*S. aethiopicum*	3	267.8 ± 34.3	4.86 ± 0.81	37.7 ± 2.4	0.46 ± 0.09	37.7 ± 3.3	4.15 ± 0.09
*S. purpureosericeum*	10	258.6 ± 15.6	4.55 ± 0.41	36.0 ± 1.3	0.59 ± 0.05	37.0 ± 1.5	3.95 ± 0.17
*S. halepense*	6	263.7 ± 20.5	4.46 ± 0.85	37.9 ± 1.5	0.45 ± 0.07	36.4 ± 2.6	3.78 ± 0.20
*S. virgatum*	1	246.3	3.46	29.2	0.57	32.7	4.76
*Sorghum* sp.	7	252.4 ± 11.4	3.62 ± 0.17	38.3 ± 1.7	0.46 ± 0.04	36.4 ± 1.8	4.47 ± 0.17
*p* (taxon effect)		0.93	0.36	0.38	0.17	0.97	0.22

Means are of genotype means; SE not shown for S. virgatum (n = 1). p-values are from one-way ANOVA across taxa represented by ≥ 2 accessions; S. virgatum was excluded from the test. **Figure 2** should display the number of accessions per taxon.

**Figure 3:**
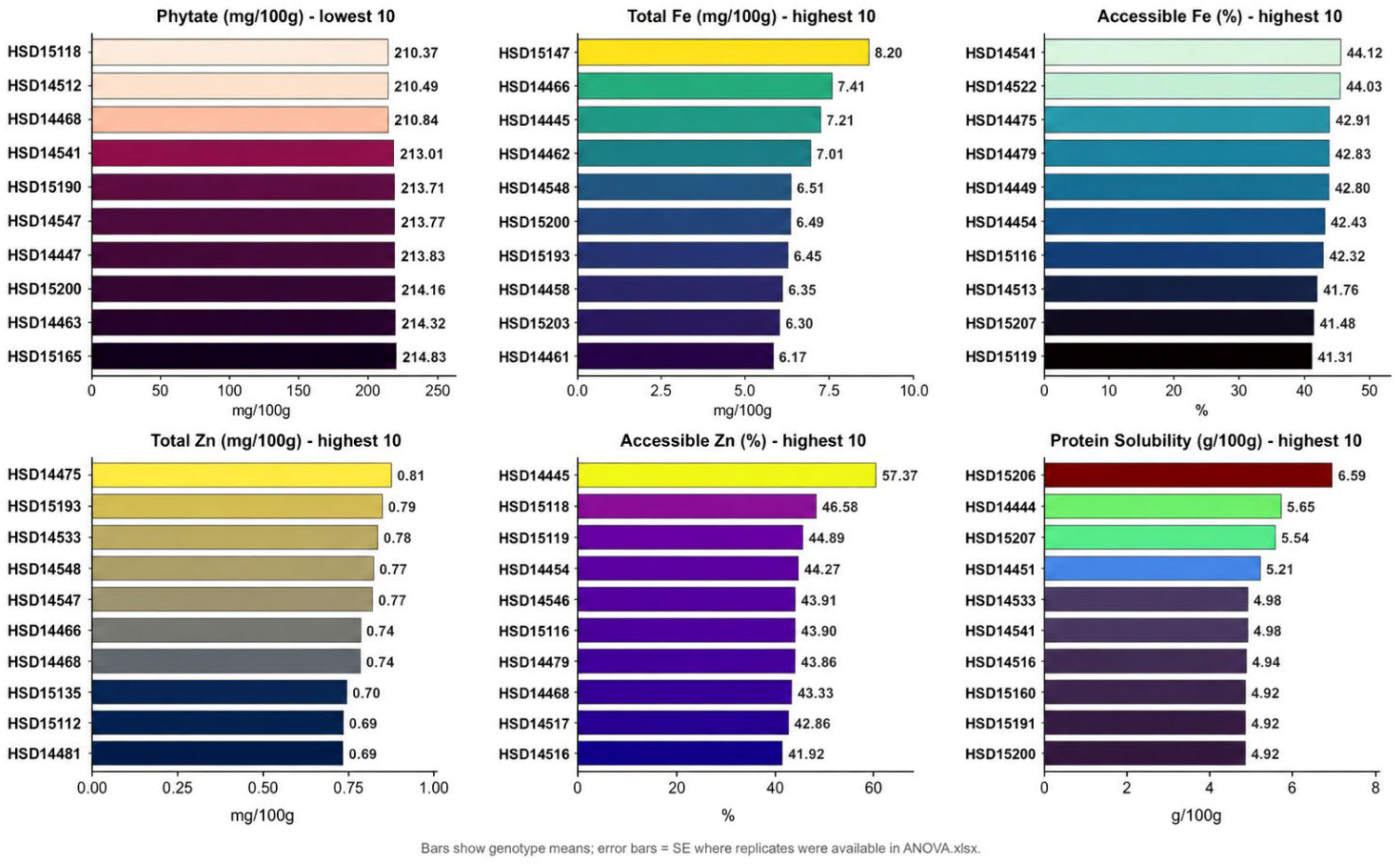
Top-performing sorghum genotypes for six nutritional traits based on mean values (± SE). Values are expressed as mean ± SE (n = 3). The traits included extractable Fe and Zn (%), phytate (mg/100 g), protein solubility (g/100 g), and total Fe and Zn (mg/100 g, dry weight).

Clear genotype-specific variations were observed across all six nutritional traits (**Figure 3**), and consistent ranking patterns were evident among several accessions. Total Fe ranged from 2.65 to 8.20 mg/100 g (mean 4.55), with HSD15147 and HSD14466 the highest-ranking accessions. Total Zn ranged from 0.25 to 0.81 mg/100 g (mean 0.52), led by HSD14475 and HSD15193 (**Figure 3**). Complete rankings for all accessions are provided in **Supplementary Tables 2b** and **S2d**.

A similar pattern was observed for the extractability traits. HCl-extractable Fe ranged from 25.65% to 44.12% (mean 35.95%), highest in HSD14541 and HSD14522. HCl-extractable Zn ranged from 29.29% to 57.37% (mean 36.29%); HSD14445 (57.37%) stood clearly apart from the rest of the panel, with the next tier near 44–47% (**Figure 3**). Full rankings are given in **Supplementary Tables 2c** and **S2e**.

Phytate ranged from 210.37 to 380.21 mg/100 g (mean 263.42). Because lower phytate is preferred for mineral extractability, the most favorable accessions were those with the lowest values, led by HSD15118, HSD14512, and HSD14468. Soluble protein ranged from 3.01 to 6.59 g/100 g (mean 4.21), highest in HSD15206 and HSD14444 (**Figure 3**). **Supplementary Tables 2a** and **S2f** give the complete rankings.

Taken together, and based strictly on the co-occurrence of low phytate with elevated protein solubility and high Fe and Zn extractability, several accessions emerged as consistent candidates across multiple traits. HSD14541 ranked among the top 10 for low phytate, extractable Fe, and protein solubility; HSD14468 combined low phytate with top-10 total and extractable Zn; HSD15200 combined low phytate with top-10 total Fe and protein solubility; and HSD15118 paired the lowest phytate value of the panel with the second-highest extractable Zn. HSD14475 linked the highest total Zn with one of the highest extractable Fe values, while HSD14479, HSD15116, and HSD14454 were jointly retained for both extractable Fe and extractable Zn. Collectively, these genotypes represent the most promising candidates for integration into sorghum nutritional improvement and biofortification breeding programs.

**Figure 4:**
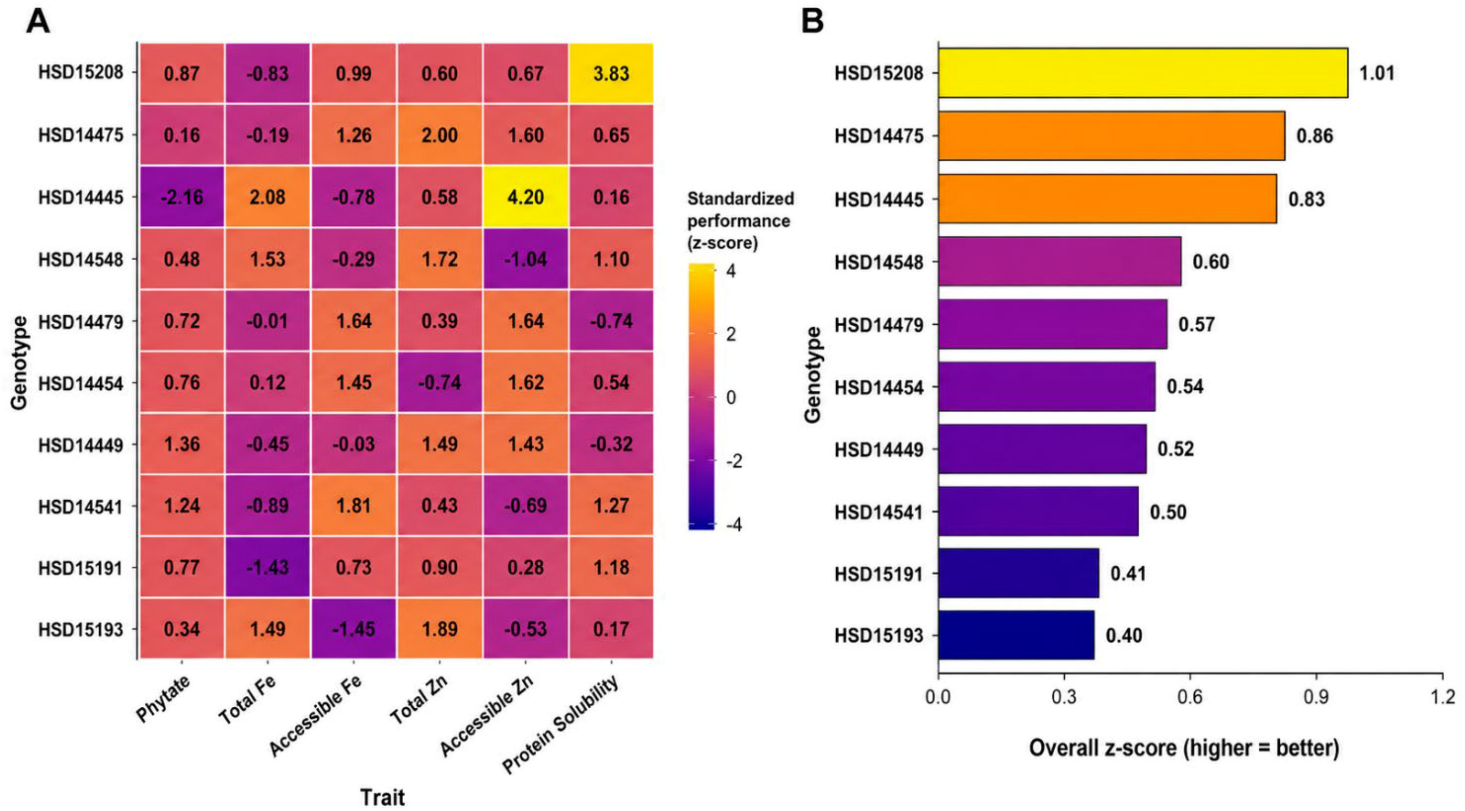
Multi-trait performance and overall ranking of the top five sorghum genotypes. **(A)** Heatmap of the standardized performance scores across the six nutritional traits for the five highest-performing genotypes. **(B)** Composite z-score ranking showing the overall dietary performance of the same genotype.

The integrated assessment of the six nutritional traits revealed clear differences among the ten top-performing sorghum genotypes (**Figure 4A**). HSD15206 ranked highest overall (z = 1.01), driven primarily by an exceptionally high protein solubility (z = 3.83), together with above-average performance for low phytate (z = 0.87), extractable Fe (z = 0.90), and total Zn (z = 0.60); its only weakness was a below-average total Fe value (z = −0.63). HSD14475 (z = 0.86) showed a markedly different profile, combining the highest total Zn of the panel (z = 2.00) with strong extractable Fe (z = 1.56), extractable Zn (z = 1.03), and protein solubility (z = 0.65). HSD14445 (z = 0.85) was distinguished by the most extreme single-trait performance in the dataset, an outstanding extractable Zn value (z = 4.28), alongside high total Fe (z = 2.08), although this was offset by a comparatively low phytate inversion score (z = −0.98) and reduced extractable Fe (z = −0.76).

Beyond the leading three, HSD14548 (z = 0.60) and HSD15193 (z = 0.40) emerged as strong total-Fe and total-Zn donors (total Fe: z = 1.53 and 1.49; total Zn: z = 1.72 and 1.86, respectively), although both displayed weaker Zn extractability. HSD14479 (z = 0.57), HSD14454 (z = 0.54), and HSD14541 (z = 0.50) clustered together as extractability-oriented genotypes, with consistently high extractable Fe (z = 1.45–1.83) and competitive extractable Zn for HSD14479 and HSD14454 (z = 1.54 and 1.62). HSD14468 (z = 0.52) combined the most favorable phytate-inversion value of the panel (z = 1.08) with high total Zn (z = 1.46) and extractable Zn (z = 1.43). HSD15191 (z = 0.41) ranked tenth, supported by elevated protein solubility (z = 1.18) and low phytate (z = 0.77), despite a weaker total Fe value (z = −1.43).

The composite z-score ranking (**Figure 4B**) reflected these patterns, with HSD15206 leading the panel (z = 1.01), closely followed by HSD14475 (z = 0.86) and HSD14445 (z = 0.85). HSD14548, HSD14479, HSD14454, HSD14468, and HSD14541 occupied an intermediate tier (z = 0.50–0.60), while HSD15191 (z = 0.41) and HSD15193 (z = 0.40) closed the top 10 ranking. Together, these accessions represent the most balanced and complementary candidates for sorghum biofortification breeding: HSD14468 and HSD14541 for low phytate; HSD14475 and HSD14548 for elevated mineral concentrations; HSD14445 and HSD14454 for superior extractability; and HSD15206 for outstanding protein solubility.

Correlation analysis among genotype means (n = 70) revealed a limited number of statistically significant associations (**Table 2**). The strongest relationship was a significant negative correlation between total Fe and extractable Fe (r = −0.53, *p* < 0.001), indicating that genotypes with higher total iron tended to show lower iron extractability. Total Fe was also positively correlated with phytate (r = 0.35, *p* = 0.003) and with total Zn (r = 0.34, *p* = 0.004), and negatively with extractable Zn (r = −0.25, *p* = 0.04). Extractable Fe and extractable Zn were positively correlated (r = 0.30, p = 0.01), and total Zn was negatively correlated with extractable Zn (r = −0.29, *p* = 0.01). By contrast, the associations between phytate and the extractable mineral fractions were weak and non-significant (phytate–extractable Fe, r = −0.19, *p* = 0.11; phytate–extractable Zn, r = −0.14, *p* = 0.23), as were all correlations involving protein solubility (r ≤ 0.11, *p* > 0.36). These non-significant relationships are not interpreted further.

**Table 2:** Pairwise Pearson correlations among six nutritional traits.

Nutritional trait	Phytate	Total Fe	HCl-extractable Fe	Total Zn	HCl-extractable Zn
Total Fe	0.35**	—			
Extractable Fe	−0.19	−0.53***	—		
Total Zn	−0.03	0.34**	−0.22	—	
Extractable Zn	−0.14	−0.25*	0.30*	−0.29*	—
Protein solubility	−0.10	−0.11	0.08	0.07	0.01

Protein-solubility correlations were all non-significant (p > 0.36). Significance: *p < 0.05, **p < 0.01, ***p < 0.001.

**Figure 5:**
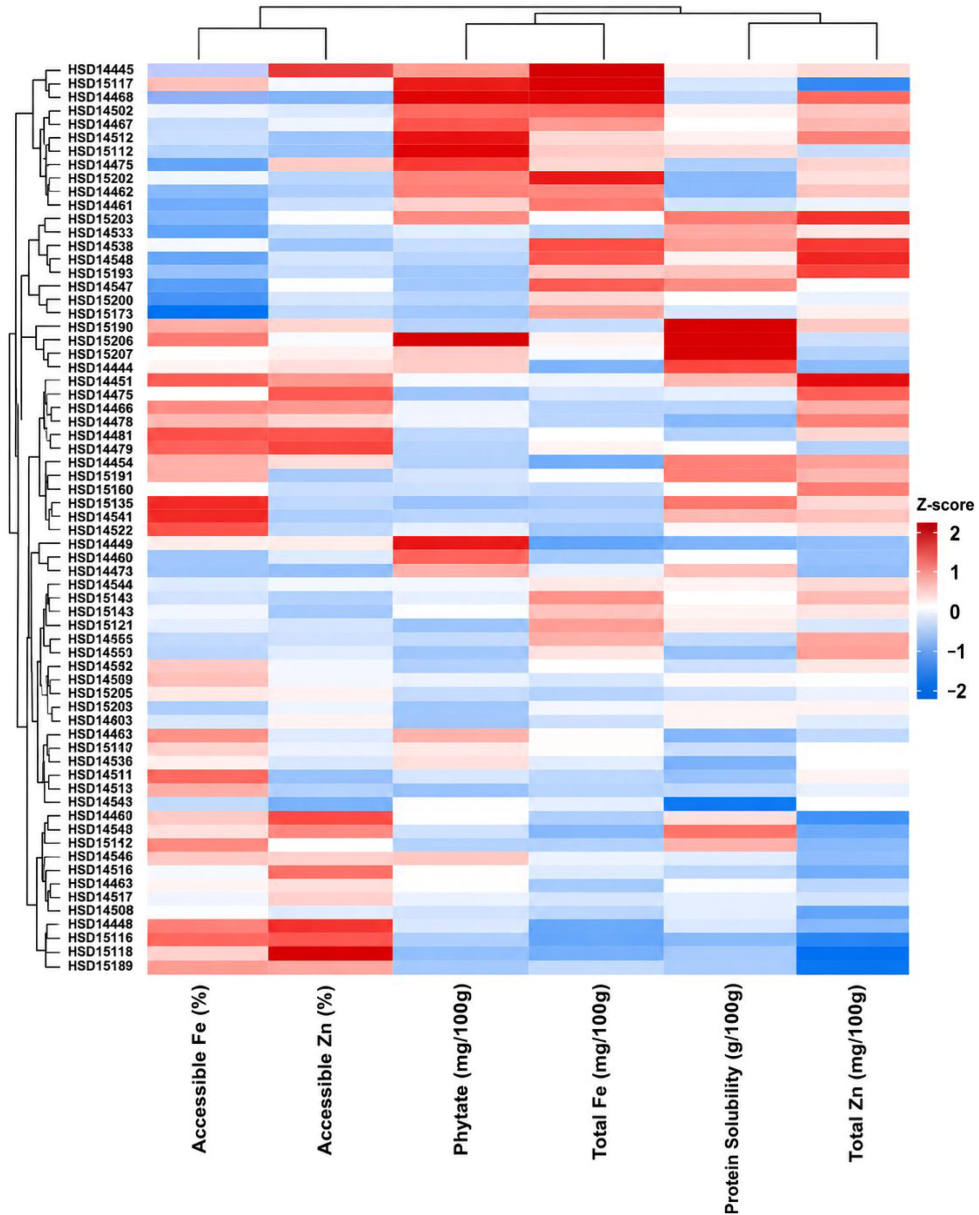
Heatmap of standardized nutritional trait profiles across sorghum genotypes.

The full-genotype nutrient heatmap revealed pronounced variation and distinct clustering across the six nutritional traits (**Figure 5**). A set of genotypes, such as HSD14445, HSD14457, HSD14468, and HSD15207, consistently showed high z-scores for mineral-related traits (extractable Zn, total Zn, and total Fe) and, in some cases, higher protein solubility. This group was designated as Cluster I (nutrient-enriched group). In contrast, genotypes HSD15189, HSD15118, and HSD15169 clustered with uniformly low z-scores across most traits, representing Cluster III (low-performing group) (**Figure 5**).

Phytate showed a contrasting pattern, varying independently of the mineral traits. Several genotypes combined relatively high mineral-related values with low phytate levels, including HSD14475, HSD14437, HSD14468, and HSD14457, defining Cluster II (low-phytate, mineral-enriched group). In comparison, other genotypes with elevated mineral traits exhibited higher phytate levels (**Figure 5**).

Hierarchical clustering separated the panel into three groups based on combined mineral and phytate profiles: Cluster I (high mineral traits with variable phytate), Cluster II (low phytate with moderate-to-high mineral levels), and Cluster III (low values across traits) (**Figure 5**).

**Figure 6:**
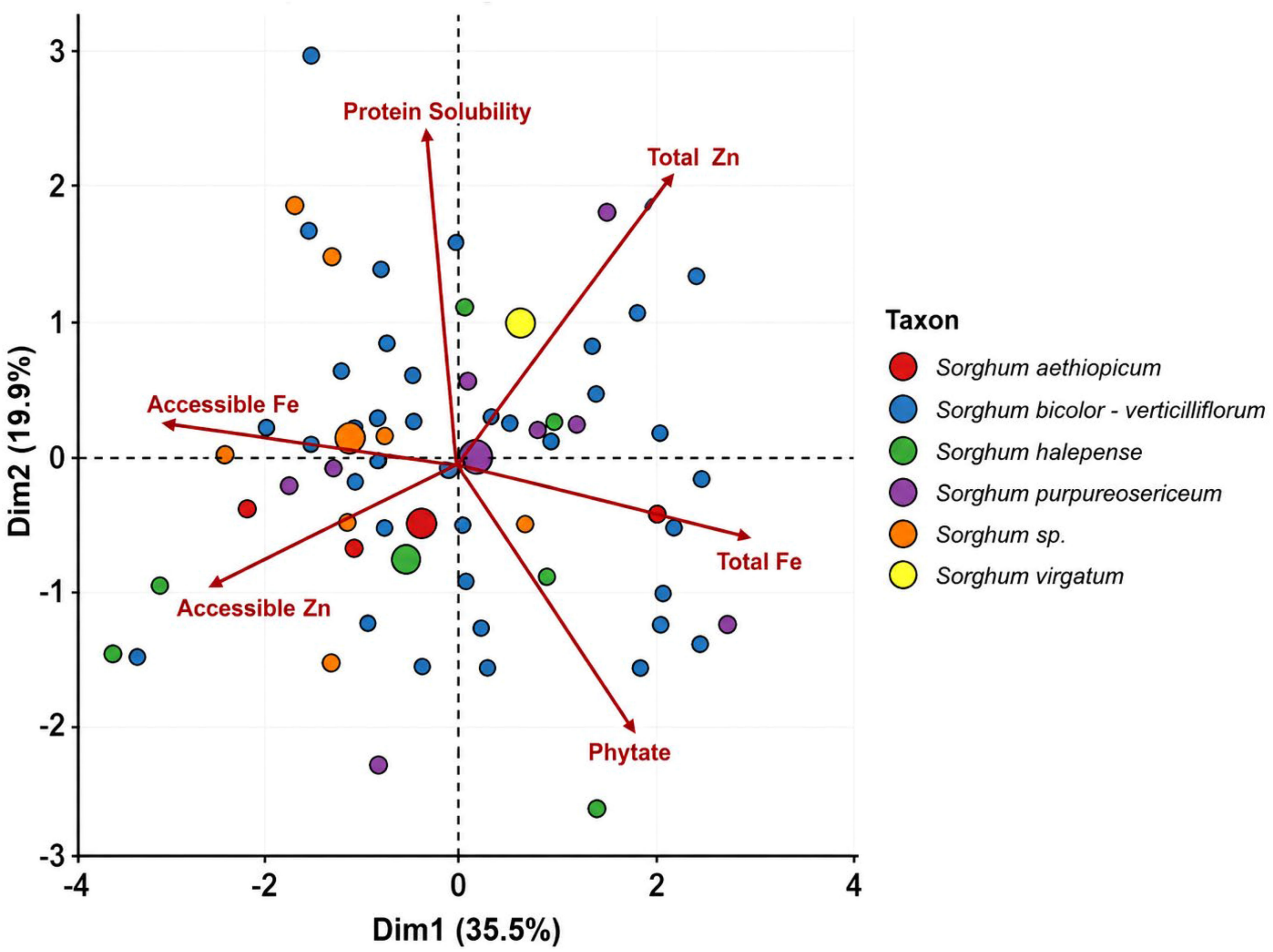
Principal component analysis (PCA) biplot of six nutritional traits of Sudanese wild sorghum genotypes. Vectors represent trait loadings for extractable Fe (%), extractable Zn (%), phytate (mg/100 g), protein solubility (g/100 g), total Zn (mg/100 g), and total Fe (mg/100 g), whereas points represent individual genotypes.

The PCA biplot revealed a clear structure of nutritional traits along the first two principal components, which together accounted for 55.4% of the total variation (Dim1 = 35.5%, Dim2 = 19.9%; **Figure 6**). Along Dim1, total Fe loaded strongly and positively, while extractable Fe and extractable Zn loaded strongly and negatively, placing the total and extractable fractions of these minerals at opposite ends of the axis. This polarity indicates an inverse relationship between mineral concentration and mineral extractability across the panel: genotypes positioned on the right of the biplot tended to combine high total Fe with low Fe and Zn extractability, whereas genotypes on the left displayed the reverse pattern.

Along Dim2, protein solubility and total Zn loaded strongly and positively, whereas phytate loaded strongly and negatively, separating these traits along the vertical axis. The near-orthogonal relationship between protein solubility and phytate suggests that variation in protein solubility is largely independent of phytate accumulation, which is desirable for biofortification breeding. The relatively large angle between total Fe and total Zn (≈ 60°) further indicates that these two mineral traits, while both positively associated with Dim1, do not co-segregate strongly across the panel, a pattern with practical implications for the simultaneous improvement of both micronutrients.

Vector orientation also clarified the pairwise relationships among traits. Extractable Fe and extractable Zn pointed in nearly the same direction (left of Dim1, slightly below the horizontal axis), confirming a strong positive co-variation between the two extractability measures. Conversely, phytate was projected to be diagonally opposite extractable Fe and extractable Zn, consistent with its established role as an anti-nutrient that limits mineral absorption: genotypes with low phytate loadings tended to be the same accessions that scored high for Fe and Zn extractability. Total Fe and phytate clustered in adjacent quadrants on the right-hand side of Dim1, suggesting that high-phytate accessions also tended to accumulate high total Fe, likely because both compounds are concentrated in the bran fraction of the kernel.

Genotype distribution in the biplot did not show strong clustering by taxon, indicating that nutritional variation within the *Sorghum bicolor* – *verticilliflorum* complex (which dominated the panel, blue points) was comparable to the variation observed across other taxa. *Sorghum aethiopicum, S. halepense*, *S. purpureosericeum*, *S. virgatum*, and the unidentified Sorghum sp. accessions, although less numerous, were dispersed across the four quadrants rather than forming compact, taxon-specific groups, suggesting that genotype-level rather than species-level selection is the most efficient strategy for identifying superior nutritional candidates in this collection (**Figure 6**).

Substantial variability was observed across the six nutritional traits (**Table 3**), with mean values ranging from 0.52 mg/100 g for total Zn to 263.42 mg/100 g for phytate. In contrast, intermediate values were recorded for total Fe (4.55 mg/100 g), extractable Fe (35.95%), extractable Zn (36.29%), and protein solubility (4.21 g/100 g).

**Table 3:** Genetic variability, broad-sense heritability, and genetic advance for six nutritional traits in 70 wild Sudanese sorghum accessions, estimated from a linear mixed model (genotype random; replicate and incomplete block within replicate as design effects) on an entry-mean basis.

Trait	Mean	GCV (%)	PCV (%)	H²	GA	GAM (%)	Genotype p
Phytate (mg/100 g)	263.42	18.70	19.04	0.96	99.78	37.88	*< 0.001*
Total Fe (mg/100 g)	4.55	27.50	28.79	0.91	2.47	54.19	*< 0.001*
Extractable Fe (%)	35.95	11.44	12.28	0.87	7.90	21.98	*< 0.001*
Total Zn (mg/100 g)	0.52	24.91	27.39	0.83	0.25	46.75	*< 0.001*
Extractable Zn (%)	36.29	12.50	13.17	0.90	8.89	24.49	*< 0.001*
Protein solubility (g/100 g)	4.21	15.59	15.64	0.99	1.35	32.04	*< 0.001*

GCV, genotypic coefficient of variation; PCV, phenotypic coefficient of variation; H², broad-sense heritability on an entry-mean basis (Vg/(Vg + Ve/r), *r* = 3); GA, expected genetic advance under selection at 5% intensity; GAM, genetic advance as a percentage of the mean. Genotype-effect p-values are from analysis of variance accounting for the alpha-lattice design. Estimates derive from a single location and season and represent an upper bound on heritability under the trial conditions.

The genotypic (GCV) and phenotypic (PCV) coefficients of variation were closely aligned across all traits, with PCV slightly exceeding GCV in each case, indicating a limited contribution of design and residual variation relative to genotypic variation under these conditions. Variability was highest for total Fe (GCV = 27.50%; PCV = 28.79%) and total Zn (GCV = 24.91%; PCV = 27.39%), followed by phytate (GCV = 18.70%; PCV = 19.04%) and protein solubility (GCV = 15.59%; PCV = 15.64%). Extractable Zn (GCV = 12.50%; PCV = 13.17%) and extractable Fe (GCV = 11.44%; PCV = 12.28%) showed comparatively lower variation (**Table 3**).

Broad-sense heritability, estimated as the ratio of genotypic to entry-mean phenotypic variance from the mixed model, was high for all traits, ranging from 0.83 for total Zn to 0.99 for protein solubility (**Table 3**), and the genotype effect was highly significant for every trait (p < 0.001). Because these estimates derive from a single location and season, the phenotypic variance does not capture genotype × environment interaction; the values therefore represent an upper bound on heritability under the trial conditions and require multi-environment validation before generalization. Expected genetic advance (GA) varied with trait scale, being largest for phytate (99.78) and smallest for total Zn (0.25); because GA is not comparable across differently scaled traits, genetic advance as a percentage of the mean (GAM) is more informative. GAM was highest for total Fe (54.19%) and total Zn (46.75%), followed by phytate (37.88%), protein solubility (32.04%), extractable Zn (24.49%), and extractable Fe (21.98%) (**Table 3**). The combination of high heritability with substantial GAM for the mineral-concentration traits suggests that additive genetic effects predominate and that direct phenotypic selection could be effective, whereas the lower GAM for the extractability traits indicates more modest expected gain

## 4 Discussion

Hidden hunger remains a major challenge in Sudan, particularly in rural areas where diets rely heavily on sorghum, locally known as *“elaish”* (“life”), underscoring its central role in ensuring food security. Despite this importance, sorghum-based diets are often deficient in essential micronutrients, contributing to persistent malnutrition and hindering progress toward Sustainable Development Goal 2.2 (More et al., 2024). Efforts to improve the nutritional quality of sorghum are constrained by the narrow genetic base of cultivated sorghum, highlighting the need to exploit the broader diversity of its wild relatives (Ananda et al., 2020; Mace et al., 2021; Venkateswaran et al., 2014).

Building on Abdelhalim et al. (2019), who first documented variation for Fe, Zn, phytate, and tannins in Sudanese wild sorghum, the present study extends this characterization by jointly profiling 70 accessions for total minerals, *in vitro* mineral accessibility, phytate, and soluble protein within a single integrated framework. This integrated phenotyping provides an early empirical basis for prioritizing accessibility-driven phenotypes alongside concentration in Sudanese wild sorghum.

Our findings revealed a clear decoupling between total Fe and Zn concentrations and their extractable fractions, demonstrating that mineral density alone is an inadequate criterion for selecting sorghum cultivars. This divergence reflects the regulatory role of grain matrix components, particularly phytate and related chelators, which govern mineral release independently of total mineral accumulation (Afify et al., 2011). Consistent with previous reports, genotypes with elevated total mineral content do not necessarily confer improved nutritional value, underscoring the limitations of conventional selection strategies based solely on mineral concentration (Frontela et al., 2011; Nsabimana et al., 2024). Therefore, these results support the integration of bioavailability proxies, such as HCl-extractable Fe and Zn, as complementary breeding traits (Guindón et al., 2021). These proxies provide a functional estimate of mineral accessibility under simulated digestive conditions and capture genotype-specific variations that are overlooked by total mineral measurements (Dwivedi et al., 2023; Singh et al., 2024). Incorporating these metrics into breeding frameworks enables a shift toward selecting nutritionally effective phenotypes, thereby improving the likelihood that genetic gains in mineral content translate into real dietary benefits (Bouis et al., 2024; Pfeiffer and McClafferty, 2007).

Our study showed a negative association between total Fe and Zn and their extractable forms. This indicates that distinct genetic mechanisms partly control mineral accumulation and their bioavailability (Clemens, 2014; Gupta et al., 2021). While the total mineral content reflects uptake and deposition, bioavailability is largely influenced by phytate levels, tissue partitioning, and transporter activity, which operate through separate genetic pathways (Dwivedi et al., 2023; Kudapa et al., 2023). Evidence across crops further shows that bioavailability traits can exhibit independent heritability and genotype × environment interactions, reinforcing the idea that higher mineral concentrations do not necessarily translate into improved nutritional value (Katuuramu et al., 2021; Roorkiwal et al., 2021).

Accordingly, improving both traits requires multi-trait or index-based selection approaches that jointly target mineral density and bioavailability (Ambrósio et al., 2024). Breeding strategies that integrate total Fe and Zn with bioavailability-related indicators, supported by marker-assisted or genomic selection, provide a practical pathway to achieve balanced gains (Stangoulis and Knez, 2022). Such frameworks are essential to ensure that genetic improvement delivers nutritionally effective outcomes rather than merely increasing concentration.

Our findings indicate that phytate contributed only weakly and non-significantly to variation in mineral extractability, implying that other grain-matrix factors predominate. This observation aligns with evidence across cereals and pseudocereals showing that, while phytate forms insoluble complexes that limit Fe and Zn absorption, it accounts for only a part of the variation observed in complex grain systems (Bohn et al., 2008; Lestienne et al., 2005; Samtiya et al., 2021). Therefore, this study supports a multifactorial model in which grain matrix composition, protein–mineral interactions, and co-occurring anti-nutrients, such as polyphenols and fibers, modulate mineral release and accessibility beyond the effects of phytate alone. For example, mineral bioavailability can remain constrained even after phytate reduction because of strong binding within the grain matrix or interactions with phenolics and structural components (Ariza‐Nieto et al., 2007; Hemalatha et al., 2006). Collectively, these results suggest that improving mineral bioavailability in sorghum will require integrative approaches that target both phytate reduction and matrix-level traits rather than relying on phytate content as a single predictive indicator.

Descriptive comparisons suggested only minor taxon-level tendencies (e.g., slightly higher total Fe in *S. aethiopicum* and higher Fe extractability in *Sorghum* spp.), none of which were statistically significant; nutritional variation in this panel was therefore predominantly genotype-specific rather than taxonomically structured. This indicates that genotype-level selection, rather than taxon-level inference, is the more reliable basis for identifying superior nutritional candidates in this collection (Paterson et al., 2019; Smith et al., 2019).

Because taxon-level differences were not statistically significant, taxonomic identity is a weak screening criterion in this panel, and prioritization is better based on individual genotype performance. Germplasm-based strategies that exploit primary and secondary gene pools remain valuable for sourcing favorable alleles, but accession-level selection rather than taxon membership should guide pre-breeding and introgression (Upadhyaya and Vetriventhan, 2018; Zhang et al., 2016). However, the effective utilization of these resources requires subsequent validation of crossability and genomic compatibility, given the reproductive barriers and linkage complexities frequently encountered between wild and cultivated gene pools (Dempewolf et al., 2014; Warschefsky et al., 2014).

Our findings showed that PCA clearly separated mineral accumulation traits from extractability and protein functionality, indicating that these traits represent distinct physiological domains rather than a single, integrated system. This separation is consistent with multivariate analyses across cereals and legumes, where mineral concentrations (e.g., Fe and Zn) load independently of phytate-related bioavailability determinants and protein-associated traits (Singh et al., 2024; Caproni et al., 2020; Guindón et al., 2021). Such domain partitioning suggests that the genetic and physiological controls underlying mineral accumulation are partly independent of those governing mineral release and utilization.

Importantly, this multivariate structure provides a practical basis for trait dissection and the design of breeding strategies, as it enables simultaneous targeted selection across domains (Altaf et al., 2024). In line with previous studies, PCA-informed trait grouping supports the development of multi-trait selection indices that can balance mineral density, extractability, and protein functionality without unintended trade-offs (Oomah et al., 2008; Shahzad et al., 2014). Therefore, the observed separation reinforces the need for integrative breeding approaches that treat these traits as coordinated but distinct targets within sorghum nutritional improvement programs.

Our findings indicated wide genotypic ranges for total and extractable Fe and Zn, phytic acid, and soluble protein, confirming substantial variability both within and across Sudanese wild sorghum taxa. This pattern is consistent with previous reports highlighting the high genetic diversity and heritability of nutritional and anti-nutritional traits in wild sorghum germplasm, supporting their value as reservoirs of favorable alleles (Abdelhalim et al., 2019; Upadhyaya and Vetriventhan, 2018). Although variation was present both within and among taxa, the among-taxon component was not statistically significant, indicating that nutritional variation in this panel is expressed predominantly at the genotype level.

Importantly, because superior nutritional profiles were not concentrated in particular taxa, effective biofortification in this germplasm should rely on genotype-level selection across the whole collection rather than on taxon-based pre-screening. Broad exploration of the wild gene pool remains valuable for capturing favorable alleles, but selection is most reliably applied at the accession level to optimize both mineral concentration and extractability (Kumar et al., 2018; V. Sharma et al., 2021; Xu et al., 2024). Therefore, integrating diversity exploration with within-taxon selection provides a robust pathway for accelerating nutritional improvement in sorghum breeding programs.

Our findings indicate that high broad-sense heritability combined with trait-specific genetic advance reflects strong genetic control and differing selection potentials among nutritional traits. The consistently high broad-sense heritabilities (0.83–0.99) confirm that most of the variation is genetically determined under these conditions, as widely reported for micronutrient and phytate traits (Caproni et al., 2020; Govindaraj et al., 2011; Guindón et al., 2021).

These results suggest that additive effects largely govern phytate and mineral extractability and that these effects can be improved through direct selection. In contrast, total mineral concentration may require genomic-assisted approaches (Roy et al., 2022). This aligns with biofortification studies that emphasize the combined use of heritability and genetic advances to prioritize traits (Mishra et al., 2022; Senguttuvel et al., 2023). Accordingly, prioritizing traits with both high heritability and genetic advance can accelerate nutritional improvement, whereas traits with lower gains may require multi-environment evaluation and molecular breeding strategies (Sinha et al., 2021).

Our findings identified Sudanese wild sorghum genotypes, such as HSD14541 and HSD14468, as promising pre-breeding materials because they combine low phytate content with high mineral extractability. This supports the role of wild sorghum as a valuable source of alleles for improving its nutritional quality (Abdelhalim et al., 2019). These genotypes provide efficient entry points for introgression, enabling the simultaneous improvement of mineral content and extractability, and supporting targeted pre-breeding strategies that integrate wild diversity into elite backgrounds for multi-trait enhancement (Ananda et al., 2020; Venkateswaran et al., 2014).

There is an urgent need to validate these promising Sudanese wild sorghum genotypes across diverse environments to ensure trait stability and relevance to breeding. Although these genotypes showed favorable profiles for total and extractable Fe and Zn, low phytic acid, and soluble protein, their expression can be influenced by genotype × environment interactions, particularly for micronutrients such as Fe (Djanaguiraman et al., 2024; Phuke et al., 2017). Therefore, multi-location evaluation is essential for identifying genotypes with consistent nutritional performance and for supporting their effective use in breeding programs, in line with biofortification strategies that integrate multi-trait phenotyping and G×E analysis (Dwivedi et al., 2023; Kudapa et al., 2023).

The clear phenotypic structuring revealed by PCA and clustering provides a strong avenue for the next phase of research, in which phenotyping is tightly integrated with genomics (Nasim et al., 2025; Roorkiwal et al., 2021). Rather than treating traits independently, this structured variation can be exploited to design more powerful GWAS and QTL mapping strategies, particularly for complex interactions, such as mineral–phytate relationships. By aligning phenotypic domains with genomic data, it is possible to resolve candidate loci with greater precision and capture coordinated trait architecture.

Looking forward, combining multi-trait phenotyping with multi-environment genomic analyses will be critical to account for both genetic effects and genotype × environment interactions. Such integration will strengthen genomic selection models and enable more reliable predictions of nutritionally superior genotypes. Ultimately, this approach provides a pathway toward more efficient, data-driven breeding strategies for improving mineral bioavailability and overall grain quality in sorghum.

## 5 Conclusion

This study demonstrates that Sudanese wild sorghum constitutes a rich and underexploited reservoir of nutritionally relevant variation, in which mineral concentration, extractability, and protein functionality are structured, yet partially independent traits. The observed decoupling between total Fe and Zn and their extractable fractions confirms that mineral density alone is an inadequate selection criterion, reinforcing the need to shift toward extractability-driven breeding. Importantly, the identification of genotypes combining low phytate with enhanced mineral extractability and favorable protein solubility provides immediate opportunities for pre-breeding and introgression. The high heritability and substantial genetic advances observed across traits further indicate strong genetic control and practical selection potential. Collectively, these findings reposition wild sorghum as a source of diversity and a functional resource for developing nutritionally effective cultivars to address micronutrient deficiencies in sorghum-based food products.

## Data Availability

The raw data supporting the conclusions of this article will be made available by the authors, without undue reservation.
