# Supplementary material for: Reframing sorghum biofortification: multi-trait nutritional dissection of Sudanese wild germplasm reveals extractability-driven breeding targets
Source: Front Plant Sci. 2026 Jul 30;17:1888511. doi: 10.3389/fpls.2026.1888511 (PMC13469446; doi:10.3389/fpls.2026.1888511)
Supplement: Supplementary file 1 [file Table1.docx]

**Supplementary Material**

Full per-accession nutritional values and trait rankings

These tables provide the complete per-accession data and rankings for all 70 wild Sudanese sorghum accessions, relocated from the main Results. In the main text, only the trait ranges, means, and a small number of notable genotypes are retained; the complete rankings are given here. Values are genotype means across three replicates. For phytate, lower values are more desirable, so Table S4a is ranked from lowest to highest; all other traits are ranked from highest to lowest.

# **Table S1. Complete nutritional profile of all accessions**

**Table S3.** Genotype means for six nutritional traits in 70 wild Sudanese sorghum accessions, listed alphabetically by accession. Values are means of three replicates.

| **Accession** | **Taxon** | **Phytate** | **Total Fe** | **Ext. Fe** | **Total Zn** | **Ext. Zn** | **Protein** |
| --- | --- | --- | --- | --- | --- | --- | --- |
| HSD14444 | *S. bicolor-vert.* | 293.70 | 4.41 | 36.10 | 0.42 | 37.44 | 5.65 |
| HSD14445 | *S. bicolor-vert.* | 314.19 | 7.21 | 32.74 | 0.58 | 57.37 | 4.27 |
| HSD14446 | *S. aethiopicum* | 240.63 | 3.57 | 35.73 | 0.33 | 34.52 | 3.98 |
| HSD14447 | *S. bicolor-vert.* | 213.83 | 3.74 | 34.16 | 0.47 | 37.14 | 4.27 |
| HSD14448 | *S. bicolor-vert.* | 252.04 | 3.90 | 35.21 | 0.46 | 38.75 | 3.98 |
| HSD14449 | *S. bicolor-vert.* | 250.17 | 3.35 | 42.80 | 0.57 | 32.87 | 4.24 |
| HSD14451 | *S. bicolor-vert.* | 292.12 | 2.71 | 36.49 | 0.37 | 38.17 | 5.21 |
| HSD14454 | *S. aethiopicum* | 226.76 | 4.68 | 42.43 | 0.42 | 44.27 | 4.21 |
| HSD14457 | *S. bicolor-vert.* | 343.65 | 6.00 | 32.74 | 0.64 | 34.80 | 4.21 |
| HSD14458 | *S. aethiopicum* | 335.88 | 6.35 | 34.82 | 0.63 | 34.33 | 4.25 |
| HSD14460 | *S. purpureosericeum* | 358.92 | 2.65 | 36.62 | 0.37 | 37.44 | 3.39 |
| HSD14461 | *S. purpureosericeum* | 333.23 | 6.17 | 30.45 | 0.61 | 31.83 | 3.39 |
| HSD14462 | *S. bicolor-vert.* | 331.76 | 7.01 | 34.48 | 0.58 | 32.15 | 3.39 |
| HSD14463 | *S. purpureosericeum* | 214.32 | 4.30 | 32.33 | 0.55 | 35.38 | 4.26 |
| HSD14466 | *S. bicolor-vert.* | 375.49 | 7.41 | 30.15 | 0.74 | 29.29 | 3.69 |
| HSD14467 | *S. bicolor-vert.* | 294.21 | 4.23 | 38.51 | 0.37 | 38.53 | 3.98 |
| HSD14468 | *S. bicolor-vert.* | 210.84 | 3.94 | 35.75 | 0.74 | 43.33 | 3.98 |
| HSD14473 | *S. bicolor-vert.* | 333.85 | 3.35 | 31.65 | 0.37 | 34.56 | 4.17 |
| HSD14475 | *S. bicolor-vert.* | 260.34 | 4.28 | 42.91 | 0.81 | 41.39 | 4.59 |
| HSD14476 | *S. bicolor-vert.* | 380.21 | 5.50 | 32.15 | 0.45 | 30.82 | 4.39 |
| HSD14478 | *S. purpureosericeum* | 255.92 | 3.57 | 40.76 | 0.64 | 41.06 | 3.71 |
| HSD14479 | *S. purpureosericeum* | 228.90 | 4.48 | 42.83 | 0.58 | 43.86 | 3.71 |
| HSD14481 | *S. purpureosericeum* | 255.64 | 3.55 | 39.43 | 0.69 | 38.50 | 3.42 |
| HSD14508 | *Sorghum sp.* | 267.36 | 3.32 | 36.65 | 0.44 | 38.21 | 4.13 |
| HSD14509 | *Sorghum sp.* | 251.87 | 3.90 | 38.61 | 0.52 | 35.54 | 4.22 |
| HSD14510 | *S. bicolor-vert.* | 303.56 | 4.60 | 40.75 | 0.42 | 34.22 | 3.36 |
| HSD14511 | *S. bicolor-vert.* | 281.90 | 4.10 | 36.85 | 0.52 | 34.18 | 3.36 |
| HSD14512 | *S. bicolor-vert.* | 210.49 | 3.55 | 39.38 | 0.49 | 32.22 | 3.76 |
| HSD14513 | *S. bicolor-vert.* | 241.42 | 3.58 | 41.76 | 0.55 | 31.05 | 3.49 |
| HSD14516 | *Sorghum sp.* | 237.36 | 2.99 | 37.54 | 0.33 | 41.92 | 4.94 |
| HSD14517 | *Sorghum sp.* | 267.37 | 4.08 | 35.66 | 0.34 | 42.86 | 3.76 |
| HSD14522 | *Sorghum sp.* | 227.18 | 3.52 | 44.03 | 0.62 | 32.19 | 4.64 |
| HSD14523 | *S. bicolor-vert.* | 223.51 | 3.47 | 40.87 | 0.36 | 36.18 | 4.64 |
| HSD14533 | *S. bicolor-vert.* | 320.56 | 4.41 | 30.18 | 0.78 | 35.81 | 4.98 |
| HSD14538 | *S. virgatum* | 246.28 | 3.46 | 29.19 | 0.57 | 32.66 | 4.76 |
| HSD14541 | *Sorghum sp.* | 213.01 | 3.38 | 44.12 | 0.59 | 32.90 | 4.98 |
| HSD14544 | *Sorghum sp.* | 302.95 | 4.18 | 31.62 | 0.37 | 30.86 | 4.59 |
| HSD14546 | *S. purpureosericeum* | 262.44 | 3.37 | 38.22 | 0.30 | 43.91 | 4.44 |
| HSD14547 | *S. purpureosericeum* | 213.77 | 5.35 | 31.35 | 0.77 | 33.12 | 4.63 |
| HSD14548 | *S. purpureosericeum* | 230.78 | 6.51 | 34.83 | 0.77 | 31.21 | 4.81 |
| HSD14550 | *Unassigned* | 217.52 | 4.94 | 33.07 | 0.66 | 34.61 | 3.48 |
| HSD14551 | *S. purpureosericeum* | 232.06 | 5.52 | 33.29 | 0.65 | 33.78 | 3.73 |
| HSD14754 | *Unassigned* | 240.05 | 4.42 | 37.07 | 0.33 | 35.58 | 4.81 |
| HSD14759 | *Unassigned* | 221.71 | 4.09 | 35.43 | 0.44 | 37.32 | 6.04 |
| HSD15112 | *S. bicolor-vert.* | 361.79 | 5.36 | 32.89 | 0.69 | 30.81 | 4.27 |
| HSD15116 | *S. halepense* | 216.80 | 2.71 | 42.32 | 0.27 | 43.90 | 3.42 |
| HSD15118 | *S. bicolor-vert.* | 210.37 | 2.84 | 38.23 | 0.25 | 46.58 | 3.62 |
| HSD15119 | *S. halepense* | 242.82 | 2.75 | 41.31 | 0.36 | 44.89 | 3.94 |
| HSD15121 | *S. halepense* | 263.11 | 5.35 | 35.26 | 0.57 | 31.91 | 4.27 |
| HSD15135 | *S. halepense* | 236.38 | 3.62 | 36.26 | 0.70 | 33.24 | 4.21 |
| HSD15136 | *S. bicolor-vert.* | 278.44 | 4.60 | 38.06 | 0.53 | 35.02 | 3.79 |
| HSD15140 | *S. bicolor-vert.* | 256.48 | 4.87 | 34.27 | 0.58 | 35.54 | 4.27 |
| HSD15143 | *S. bicolor-vert.* | 250.10 | 5.80 | 33.90 | 0.62 | 32.21 | 4.21 |
| HSD15147 | *S. halepense* | 360.02 | 8.20 | 39.31 | 0.27 | 35.22 | 3.86 |
| HSD15152 | *S. bicolor-vert.* | 220.98 | 4.58 | 38.37 | 0.57 | 35.68 | 3.84 |
| HSD15156 | *S. halepense* | 263.19 | 4.13 | 33.22 | 0.53 | 29.42 | 3.01 |
| HSD15160 | *S. bicolor-vert.* | 238.58 | 4.46 | 39.30 | 0.63 | 31.51 | 4.93 |
| HSD15165 | *S. bicolor-vert.* | 214.83 | 5.67 | 34.97 | 0.48 | 34.02 | 4.29 |
| HSD15173 | *S. bicolor-vert.* | 224.93 | 5.08 | 29.15 | 0.49 | 34.06 | 4.21 |
| HSD15189 | *S. bicolor-vert.* | 221.47 | 3.71 | 40.05 | 0.28 | 40.04 | 3.67 |
| HSD15190 | *S. bicolor-vert.* | 213.71 | 5.74 | 25.65 | 0.56 | 32.29 | 3.87 |
| HSD15191 | *S. bicolor-vert.* | 226.20 | 2.67 | 39.49 | 0.66 | 37.69 | 4.93 |
| HSD15193 | *S. bicolor-vert.* | 222.85 | 6.45 | 29.72 | 0.79 | 33.71 | 4.29 |
| HSD15199 | *S. bicolor-vert.* | 212.82 | 6.76 | 26.10 | 0.51 | 34.48 | 3.82 |
| HSD15200 | *S. bicolor-vert.* | 214.16 | 6.49 | 29.28 | 0.52 | 36.03 | 4.93 |
| HSD15202 | *S. bicolor-vert.* | 350.16 | 5.35 | 29.46 | 0.58 | 39.24 | 3.61 |
| HSD15203 | *S. bicolor-vert.* | 297.23 | 6.30 | 29.79 | 0.49 | 33.08 | 3.82 |
| HSD15205 | *S. bicolor-vert.* | 233.14 | 3.46 | 37.03 | 0.49 | 37.08 | 3.82 |
| HSD15206 | *S. bicolor-vert.* | 221.47 | 3.71 | 40.05 | 0.61 | 38.64 | 6.59 |
| HSD15207 | *S. bicolor-vert.* | 377.75 | 4.67 | 41.48 | 0.45 | 35.69 | 5.54 |

*Phytate (mg/100 g); Total Fe (mg/100 g); Ext. Fe = HCl-extractable Fe (%); Total Zn (mg/100 g); Ext. Zn = HCl-extractable Zn (%); Protein = soluble protein (g/100 g). Taxon abbreviations: S. bicolor-vert. = S. bicolor subsp. verticilliflorum.*

# **Table S2. Full rankings of all accessions, by trait**

**Table S2a.** Phytate (mg/100 g) ranked from lowest (most desirable) to highest (n = 70).

| **Rank** | **Accession** | **Value** | **Rank** | **Accession** | **Value** |
| --- | --- | --- | --- | --- | --- |
| 1 | HSD15118 | 210.37 | 36 | HSD15143 | 250.10 |
| 2 | HSD14512 | 210.49 | 37 | HSD14449 | 250.17 |
| 3 | HSD14468 | 210.84 | 38 | HSD14509 | 251.87 |
| 4 | HSD15199 | 212.82 | 39 | HSD14448 | 252.04 |
| 5 | HSD14541 | 213.01 | 40 | HSD14481 | 255.64 |
| 6 | HSD15190 | 213.71 | 41 | HSD14478 | 255.92 |
| 7 | HSD14547 | 213.77 | 42 | HSD15140 | 256.48 |
| 8 | HSD14447 | 213.83 | 43 | HSD14475 | 260.34 |
| 9 | HSD15200 | 214.16 | 44 | HSD14546 | 262.44 |
| 10 | HSD14463 | 214.32 | 45 | HSD15121 | 263.11 |
| 11 | HSD15165 | 214.83 | 46 | HSD15156 | 263.19 |
| 12 | HSD15116 | 216.80 | 47 | HSD14508 | 267.36 |
| 13 | HSD14550 | 217.52 | 48 | HSD14517 | 267.37 |
| 14 | HSD15152 | 220.98 | 49 | HSD15136 | 278.44 |
| 15 | HSD15206 | 221.47 | 50 | HSD14511 | 281.90 |
| 16 | HSD15189 | 221.47 | 51 | HSD14451 | 292.12 |
| 17 | HSD14759 | 221.71 | 52 | HSD14444 | 293.70 |
| 18 | HSD15193 | 222.85 | 53 | HSD14467 | 294.21 |
| 19 | HSD14523 | 223.51 | 54 | HSD15203 | 297.23 |
| 20 | HSD15173 | 224.93 | 55 | HSD14544 | 302.95 |
| 21 | HSD15191 | 226.20 | 56 | HSD14510 | 303.56 |
| 22 | HSD14454 | 226.76 | 57 | HSD14445 | 314.19 |
| 23 | HSD14522 | 227.18 | 58 | HSD14533 | 320.56 |
| 24 | HSD14479 | 228.90 | 59 | HSD14462 | 331.76 |
| 25 | HSD14548 | 230.78 | 60 | HSD14461 | 333.23 |
| 26 | HSD14551 | 232.06 | 61 | HSD14473 | 333.85 |
| 27 | HSD15205 | 233.14 | 62 | HSD14458 | 335.88 |
| 28 | HSD15135 | 236.38 | 63 | HSD14457 | 343.65 |
| 29 | HSD14516 | 237.36 | 64 | HSD15202 | 350.16 |
| 30 | HSD15160 | 238.58 | 65 | HSD14460 | 358.92 |
| 31 | HSD14754 | 240.05 | 66 | HSD15147 | 360.02 |
| 32 | HSD14446 | 240.63 | 67 | HSD15112 | 361.79 |
| 33 | HSD14513 | 241.42 | 68 | HSD14466 | 375.49 |
| 34 | HSD15119 | 242.82 | 69 | HSD15207 | 377.75 |
| 35 | HSD14538 | 246.28 | 70 | HSD14476 | 380.21 |

**Table S2b.** Total Fe (mg/100 g) ranked from highest to lowest (n = 70).

| **Rank** | **Accession** | **Value** | **Rank** | **Accession** | **Value** |
| --- | --- | --- | --- | --- | --- |
| 1 | HSD15147 | 8.20 | 36 | HSD14475 | 4.28 |
| 2 | HSD14466 | 7.41 | 37 | HSD14467 | 4.23 |
| 3 | HSD14445 | 7.21 | 38 | HSD14544 | 4.18 |
| 4 | HSD14462 | 7.01 | 39 | HSD15156 | 4.13 |
| 5 | HSD15199 | 6.76 | 40 | HSD14511 | 4.10 |
| 6 | HSD14548 | 6.51 | 41 | HSD14759 | 4.09 |
| 7 | HSD15200 | 6.49 | 42 | HSD14517 | 4.08 |
| 8 | HSD15193 | 6.45 | 43 | HSD14468 | 3.94 |
| 9 | HSD14458 | 6.35 | 44 | HSD14509 | 3.90 |
| 10 | HSD15203 | 6.30 | 45 | HSD14448 | 3.90 |
| 11 | HSD14461 | 6.17 | 46 | HSD14447 | 3.74 |
| 12 | HSD14457 | 6.00 | 47 | HSD15206 | 3.71 |
| 13 | HSD15143 | 5.80 | 48 | HSD15189 | 3.71 |
| 14 | HSD15190 | 5.74 | 49 | HSD15135 | 3.62 |
| 15 | HSD15165 | 5.67 | 50 | HSD14513 | 3.58 |
| 16 | HSD14551 | 5.52 | 51 | HSD14478 | 3.57 |
| 17 | HSD14476 | 5.50 | 52 | HSD14446 | 3.57 |
| 18 | HSD15112 | 5.36 | 53 | HSD14481 | 3.55 |
| 19 | HSD14547 | 5.35 | 54 | HSD14512 | 3.55 |
| 20 | HSD15121 | 5.35 | 55 | HSD14522 | 3.52 |
| 21 | HSD15202 | 5.35 | 56 | HSD14523 | 3.47 |
| 22 | HSD15173 | 5.08 | 57 | HSD14538 | 3.46 |
| 23 | HSD14550 | 4.94 | 58 | HSD15205 | 3.46 |
| 24 | HSD15140 | 4.87 | 59 | HSD14541 | 3.38 |
| 25 | HSD14454 | 4.68 | 60 | HSD14546 | 3.37 |
| 26 | HSD15207 | 4.67 | 61 | HSD14449 | 3.35 |
| 27 | HSD14510 | 4.60 | 62 | HSD14473 | 3.35 |
| 28 | HSD15136 | 4.60 | 63 | HSD14508 | 3.32 |
| 29 | HSD15152 | 4.58 | 64 | HSD14516 | 2.99 |
| 30 | HSD14479 | 4.48 | 65 | HSD15118 | 2.84 |
| 31 | HSD15160 | 4.46 | 66 | HSD15119 | 2.75 |
| 32 | HSD14754 | 4.42 | 67 | HSD15116 | 2.71 |
| 33 | HSD14444 | 4.41 | 68 | HSD14451 | 2.71 |
| 34 | HSD14533 | 4.41 | 69 | HSD15191 | 2.67 |
| 35 | HSD14463 | 4.30 | 70 | HSD14460 | 2.65 |

**Table S2c.** HCl-extractable Fe (%), ranked from highest to lowest (n = 70).

| **Rank** | **Accession** | **Value** | **Rank** | **Accession** | **Value** |
| --- | --- | --- | --- | --- | --- |
| 1 | HSD14541 | 44.12 | 36 | HSD14468 | 35.75 |
| 2 | HSD14522 | 44.03 | 37 | HSD14446 | 35.73 |
| 3 | HSD14475 | 42.91 | 38 | HSD14517 | 35.66 |
| 4 | HSD14479 | 42.83 | 39 | HSD14759 | 35.43 |
| 5 | HSD14449 | 42.80 | 40 | HSD15121 | 35.26 |
| 6 | HSD14454 | 42.43 | 41 | HSD14448 | 35.21 |
| 7 | HSD15116 | 42.32 | 42 | HSD15165 | 34.97 |
| 8 | HSD14513 | 41.76 | 43 | HSD14548 | 34.83 |
| 9 | HSD15207 | 41.48 | 44 | HSD14458 | 34.82 |
| 10 | HSD15119 | 41.31 | 45 | HSD14462 | 34.48 |
| 11 | HSD14523 | 40.87 | 46 | HSD15140 | 34.27 |
| 12 | HSD14478 | 40.76 | 47 | HSD14447 | 34.16 |
| 13 | HSD14510 | 40.75 | 48 | HSD15143 | 33.90 |
| 14 | HSD15189 | 40.05 | 49 | HSD14551 | 33.29 |
| 15 | HSD15206 | 40.05 | 50 | HSD15156 | 33.22 |
| 16 | HSD15191 | 39.49 | 51 | HSD14550 | 33.07 |
| 17 | HSD14481 | 39.43 | 52 | HSD15112 | 32.89 |
| 18 | HSD14512 | 39.38 | 53 | HSD14445 | 32.74 |
| 19 | HSD15147 | 39.31 | 54 | HSD14457 | 32.74 |
| 20 | HSD15160 | 39.30 | 55 | HSD14463 | 32.33 |
| 21 | HSD14509 | 38.61 | 56 | HSD14476 | 32.15 |
| 22 | HSD14467 | 38.51 | 57 | HSD14473 | 31.65 |
| 23 | HSD15152 | 38.37 | 58 | HSD14544 | 31.62 |
| 24 | HSD15118 | 38.23 | 59 | HSD14547 | 31.35 |
| 25 | HSD14546 | 38.22 | 60 | HSD14461 | 30.45 |
| 26 | HSD15136 | 38.06 | 61 | HSD14533 | 30.18 |
| 27 | HSD14516 | 37.54 | 62 | HSD14466 | 30.15 |
| 28 | HSD14754 | 37.07 | 63 | HSD15203 | 29.79 |
| 29 | HSD15205 | 37.03 | 64 | HSD15193 | 29.72 |
| 30 | HSD14511 | 36.85 | 65 | HSD15202 | 29.46 |
| 31 | HSD14508 | 36.65 | 66 | HSD15200 | 29.28 |
| 32 | HSD14460 | 36.62 | 67 | HSD14538 | 29.19 |
| 33 | HSD14451 | 36.49 | 68 | HSD15173 | 29.15 |
| 34 | HSD15135 | 36.26 | 69 | HSD15199 | 26.10 |
| 35 | HSD14444 | 36.10 | 70 | HSD15190 | 25.65 |

**Table S2d.** Total Zn (mg/100 g) ranked from highest to lowest (n = 70).

| **Rank** | **Accession** | **Value** | **Rank** | **Accession** | **Value** |
| --- | --- | --- | --- | --- | --- |
| 1 | HSD14475 | 0.81 | 36 | HSD15156 | 0.53 |
| 2 | HSD15193 | 0.79 | 37 | HSD14511 | 0.52 |
| 3 | HSD14533 | 0.78 | 38 | HSD15200 | 0.52 |
| 4 | HSD14548 | 0.77 | 39 | HSD14509 | 0.52 |
| 5 | HSD14547 | 0.77 | 40 | HSD15199 | 0.51 |
| 6 | HSD14466 | 0.74 | 41 | HSD15205 | 0.49 |
| 7 | HSD14468 | 0.74 | 42 | HSD15203 | 0.49 |
| 8 | HSD15135 | 0.70 | 43 | HSD15173 | 0.49 |
| 9 | HSD15112 | 0.69 | 44 | HSD14512 | 0.49 |
| 10 | HSD14481 | 0.69 | 45 | HSD15165 | 0.48 |
| 11 | HSD14550 | 0.66 | 46 | HSD14447 | 0.47 |
| 12 | HSD15191 | 0.66 | 47 | HSD14448 | 0.46 |
| 13 | HSD14551 | 0.65 | 48 | HSD15207 | 0.45 |
| 14 | HSD14457 | 0.64 | 49 | HSD14476 | 0.45 |
| 15 | HSD14478 | 0.64 | 50 | HSD14759 | 0.44 |
| 16 | HSD14458 | 0.63 | 51 | HSD14508 | 0.44 |
| 17 | HSD15160 | 0.63 | 52 | HSD14444 | 0.42 |
| 18 | HSD15143 | 0.62 | 53 | HSD14454 | 0.42 |
| 19 | HSD14522 | 0.62 | 54 | HSD14510 | 0.42 |
| 20 | HSD15206 | 0.61 | 55 | HSD14467 | 0.37 |
| 21 | HSD14461 | 0.61 | 56 | HSD14473 | 0.37 |
| 22 | HSD14541 | 0.59 | 57 | HSD14451 | 0.37 |
| 23 | HSD14479 | 0.58 | 58 | HSD14544 | 0.37 |
| 24 | HSD15140 | 0.58 | 59 | HSD14460 | 0.37 |
| 25 | HSD15202 | 0.58 | 60 | HSD14523 | 0.36 |
| 26 | HSD14445 | 0.58 | 61 | HSD15119 | 0.36 |
| 27 | HSD14462 | 0.58 | 62 | HSD14517 | 0.34 |
| 28 | HSD14449 | 0.57 | 63 | HSD14516 | 0.33 |
| 29 | HSD15152 | 0.57 | 64 | HSD14446 | 0.33 |
| 30 | HSD14538 | 0.57 | 65 | HSD14754 | 0.33 |
| 31 | HSD15121 | 0.57 | 66 | HSD14546 | 0.30 |
| 32 | HSD15190 | 0.56 | 67 | HSD15189 | 0.28 |
| 33 | HSD14463 | 0.55 | 68 | HSD15116 | 0.27 |
| 34 | HSD14513 | 0.55 | 69 | HSD15147 | 0.27 |
| 35 | HSD15136 | 0.53 | 70 | HSD15118 | 0.25 |

**Table S2e.** HCl-extractable Zn (%) ranked from highest to lowest (n = 70).

| **Rank** | **Accession** | **Value** | **Rank** | **Accession** | **Value** |
| --- | --- | --- | --- | --- | --- |
| 1 | HSD14445 | 57.37 | 36 | HSD15147 | 35.22 |
| 2 | HSD15118 | 46.58 | 37 | HSD15136 | 35.02 |
| 3 | HSD15119 | 44.89 | 38 | HSD14457 | 34.80 |
| 4 | HSD14454 | 44.27 | 39 | HSD14550 | 34.61 |
| 5 | HSD14546 | 43.91 | 40 | HSD14473 | 34.56 |
| 6 | HSD15116 | 43.90 | 41 | HSD14446 | 34.52 |
| 7 | HSD14479 | 43.86 | 42 | HSD15199 | 34.48 |
| 8 | HSD14468 | 43.33 | 43 | HSD14458 | 34.33 |
| 9 | HSD14517 | 42.86 | 44 | HSD14510 | 34.22 |
| 10 | HSD14516 | 41.92 | 45 | HSD14511 | 34.18 |
| 11 | HSD14475 | 41.39 | 46 | HSD15173 | 34.06 |
| 12 | HSD14478 | 41.06 | 47 | HSD15165 | 34.02 |
| 13 | HSD15189 | 40.04 | 48 | HSD14551 | 33.78 |
| 14 | HSD15202 | 39.24 | 49 | HSD15193 | 33.71 |
| 15 | HSD14448 | 38.75 | 50 | HSD15135 | 33.24 |
| 16 | HSD15206 | 38.64 | 51 | HSD14547 | 33.12 |
| 17 | HSD14467 | 38.53 | 52 | HSD15203 | 33.08 |
| 18 | HSD14481 | 38.50 | 53 | HSD14541 | 32.90 |
| 19 | HSD14508 | 38.21 | 54 | HSD14449 | 32.87 |
| 20 | HSD14451 | 38.17 | 55 | HSD14538 | 32.66 |
| 21 | HSD15191 | 37.69 | 56 | HSD15190 | 32.29 |
| 22 | HSD14460 | 37.44 | 57 | HSD14512 | 32.22 |
| 23 | HSD14444 | 37.44 | 58 | HSD15143 | 32.21 |
| 24 | HSD14759 | 37.32 | 59 | HSD14522 | 32.19 |
| 25 | HSD14447 | 37.14 | 60 | HSD14462 | 32.15 |
| 26 | HSD15205 | 37.08 | 61 | HSD15121 | 31.91 |
| 27 | HSD14523 | 36.18 | 62 | HSD14461 | 31.83 |
| 28 | HSD15200 | 36.03 | 63 | HSD15160 | 31.51 |
| 29 | HSD14533 | 35.81 | 64 | HSD14548 | 31.21 |
| 30 | HSD15207 | 35.69 | 65 | HSD14513 | 31.05 |
| 31 | HSD15152 | 35.68 | 66 | HSD14544 | 30.86 |
| 32 | HSD14754 | 35.58 | 67 | HSD14476 | 30.82 |
| 33 | HSD15140 | 35.54 | 68 | HSD15112 | 30.81 |
| 34 | HSD14509 | 35.54 | 69 | HSD15156 | 29.42 |
| 35 | HSD14463 | 35.38 | 70 | HSD14466 | 29.29 |

**Table S2f.** Soluble protein (g/100 g) ranked from highest to lowest (n = 70).

| **Rank** | **Accession** | **Value** | **Rank** | **Accession** | **Value** |
| --- | --- | --- | --- | --- | --- |
| 1 | HSD15206 | 6.59 | 36 | HSD15135 | 4.21 |
| 2 | HSD14759 | 6.04 | 37 | HSD15143 | 4.21 |
| 3 | HSD14444 | 5.65 | 38 | HSD14473 | 4.17 |
| 4 | HSD15207 | 5.54 | 39 | HSD14508 | 4.13 |
| 5 | HSD14451 | 5.21 | 40 | HSD14467 | 3.98 |
| 6 | HSD14533 | 4.98 | 41 | HSD14468 | 3.98 |
| 7 | HSD14541 | 4.98 | 42 | HSD14446 | 3.98 |
| 8 | HSD14516 | 4.94 | 43 | HSD14448 | 3.98 |
| 9 | HSD15200 | 4.93 | 44 | HSD15119 | 3.94 |
| 10 | HSD15160 | 4.93 | 45 | HSD15190 | 3.87 |
| 11 | HSD15191 | 4.93 | 46 | HSD15147 | 3.86 |
| 12 | HSD14548 | 4.81 | 47 | HSD15152 | 3.84 |
| 13 | HSD14754 | 4.81 | 48 | HSD15203 | 3.82 |
| 14 | HSD14538 | 4.76 | 49 | HSD15205 | 3.82 |
| 15 | HSD14523 | 4.64 | 50 | HSD15199 | 3.82 |
| 16 | HSD14522 | 4.64 | 51 | HSD15136 | 3.79 |
| 17 | HSD14547 | 4.63 | 52 | HSD14512 | 3.76 |
| 18 | HSD14475 | 4.59 | 53 | HSD14517 | 3.76 |
| 19 | HSD14544 | 4.59 | 54 | HSD14551 | 3.73 |
| 20 | HSD14546 | 4.44 | 55 | HSD14479 | 3.71 |
| 21 | HSD14476 | 4.39 | 56 | HSD14478 | 3.71 |
| 22 | HSD15193 | 4.29 | 57 | HSD14466 | 3.69 |
| 23 | HSD15165 | 4.29 | 58 | HSD15189 | 3.67 |
| 24 | HSD15121 | 4.27 | 59 | HSD15118 | 3.62 |
| 25 | HSD15112 | 4.27 | 60 | HSD15202 | 3.61 |
| 26 | HSD15140 | 4.27 | 61 | HSD14513 | 3.49 |
| 27 | HSD14445 | 4.27 | 62 | HSD14550 | 3.48 |
| 28 | HSD14447 | 4.27 | 63 | HSD15116 | 3.42 |
| 29 | HSD14463 | 4.26 | 64 | HSD14481 | 3.42 |
| 30 | HSD14458 | 4.25 | 65 | HSD14462 | 3.39 |
| 31 | HSD14449 | 4.24 | 66 | HSD14461 | 3.39 |
| 32 | HSD14509 | 4.22 | 67 | HSD14460 | 3.39 |
| 33 | HSD14457 | 4.21 | 68 | HSD14511 | 3.36 |
| 34 | HSD14454 | 4.21 | 69 | HSD14510 | 3.36 |
| 35 | HSD15173 | 4.21 | 70 | HSD15156 | 3.01 |
